# Fufang Zhenzhu Tiaozhi Capsule Prevents Intestinal Inflammation and Barrier Disruption in Mice With Non-Alcoholic Steatohepatitis

**DOI:** 10.3389/fendo.2022.864703

**Published:** 2022-06-16

**Authors:** Tian Lan, Tonghao Xu, Yanfang Fu, Shuo Jiang, Xiaolin Liang, Ze Yu, Linyu Pan, Xianglu Rong, Jiao Guo

**Affiliations:** ^1^ Institute of Chinese Medicine, Guangdong Pharmaceutical University, Guangzhou, China; ^2^ Guangdong Metabolic Diseases Research Center of Integrated Chinese and Western Medicine, Guangdong Pharmaceutical University, Guangzhou, China; ^3^ Key Laboratory of Glucolipid Metabolic Disorder, Ministry of Education, Guangzhou, China; ^4^ Guangdong Traditional Chinese Medicine Key Laboratory for Metabolic Diseases, Guangdong Pharmaceutical University, Guangzhou, China

**Keywords:** NASH, gut–liver axis, intestinal barrier, intestinal microbiota, traditional Chinese medicine

## Abstract

Nonalcoholic steatohepatitis (NASH) has become a major cause of liver transplantation and liver-associated death. Targeting the gut–liver axis is a potential therapy for NASH. The Fufang Zhenzhu Tiaozhi (FTZ) capsule, a traditional Chinese medicine commonly used in clinical practice, has recently emerged as a promising drug candidate for metabolic diseases such as NASH. The present study aimed to investigate whether FTZ exerts an anti-NASH effect by targeting the gut–liver axis. Mice were fed with a high-fat diet (HFD) for 20 weeks to induce NASH. HFD-fed mice were daily intragastrically administrated with FTZ at 10 weeks after tbe initiation of HFD feeding. The mRNA levels of genes associated with the intestinal tight junction, lipid metabolism, and inflammation were determined by the q-PCR assay. Hepatic pathology was evaluated by H&E staining. The gut microbiota was analyzed by 16S rRNA gene sequencing. FTZ attenuated HFD-induced obesity, insulin resistance, and hepatic steatosis in mice. FTZ treatment decreased the elevated levels of serum aminotransferases and liver triglyceride in NASH mice. Furthermore, FTZ treatment reduced hepatic inflammatory cell infiltration and fibrosis in mice. In addition, FTZ attenuated the intestinal inflammatory response and improved intestinal barrier function. Mechanistically, FTZ-treated mice showed a different gut microbiota composition compared with that in HFD-fed mice. Finally, we identified eight differential metabolites that may contribute to the improvement of NASH with FTZ treatment. In summary, FTZ ameliorates NASH by inhibiting gut inflammation, improving intestinal barrier function, and modulating intestinal microbiota composition.

## Introduction

Nonalcoholic fatty liver disease (NAFLD) is a disease spectrum that ranges from nonalcoholic fatty liver (NAFL) to nonalcoholic steatohepatitis (NASH) characterized by progressive fibrosis (1). Hepatic steatosis and chronic low-grade inflammation caused by prolonged exposure of liver lobules to high-flux free fatty acid (FFA) lead to metabolic liver injury in patients with NASH. NASH can progress over time to irreversible cirrhosis or hepatocellular carcinoma (HCC), and there is no better treatment than liver transplantation for them (2–4). With constant lifestyle changing, the burden of NAFLD in China is accordingly increasing. From 1999 to 2018, the prevalence of NAFLD in China has increased by 8%–9%, with the total prevalence now reaching 29.1%, which makes it an essential social health issue (5). East Asian populations may have a higher risk of developing NASH due to a more frequent mutation of lipid metabolism genes (6).

The improvement of metabolic inflammation and progressive liver fibrosis caused by disorders of lipid metabolism is a major clinical therapy for NASH (7, 8). Although the pharmacology industry has invested heavily in this in recent years, there are still no approved therapeutic drugs for NASH on the market (9). At present, the only effective treatment is changing a poor lifestyle and diet or engaging in physical activity or bariatric surgery (10, 11). Therefore, there is a severe challenge for the development of drugs to treat NAFLD, particularly targeting the inflammation and progressive fibrosis of NASH.

There is growing evidence that demonstrated intestinal mucosal inflammation, intestinal flora, and barrier function are implicated in the pathogenesis of NASH (12, 13). Mice with defects in intestinal epithelial permeability developed a more severe steatohepatitis. Additionally, the clinical studies revealed that patients with NASH exerted increased gut epithelial permeability, a decreased expression of tight junction proteins, and higher levels of inflammation (14–16), contributing to the progression of NAFLD. Furthermore, NASH is closely associated with changes in the composition of the gut microbiota. Many species in human gut microbiota are thought to be associated with the progression of NAFLD such as *Ruminococcus*, *Roseburia*, and *Bacteroidia* (17). In summary, the complex interaction of intestinal flora, intestinal permeability, and inflammation-mediated “liver-gut axis” regulate the progression of NAFLD and NASH (14, 18, 19).

Fufang Zhenzhu Tiaozhi (FTZ), a traditional Chinese medicine formula for treating metabolic syndromes, is composed of eight traditional Chinese medicinal herbs, including *Rhizoma coptidis*, *Fructus Ligustri Lucidi*, *Herba cirsii japonici*, *Radix Salvia miltiorrhiza*, *Radix Notoginseng*, *Cortex Eucommiae*, *Fructus Citri Sarcodactylis*, and *Radix Atractylodes macrocephala* (20). Our previous studies have shown that FTZ effectively alleviates hyperglycemia and reduces hyperlipidemia by the regulation of cytochrome P450 family 7 subfamily A member 1 (CYP7A1), cytochrome P450 family 7 subfamily A member 1 (HMG-CoA), and insulin receptor substrate 1 (IRS1-GLUT2) (21, 22). Previous studies on FTZ for NASH tended to be less systematic and focused only on the liver, neglecting the important role played by extra-hepatic tissues such as the gut (23, 24). However, whether FTZ prevents NASH through the regulation of the liver–gut axis is still unclear.

In the present study, we demonstrated that FTZ attenuated hepatic triglyceride (TG) accumulation, hepatic inflammation, and fibrosis in mice with NASH. In addition, FTZ showed a more potent anti-NASH property than atorvastatin involved in the attenuation of hepatic inflammation and fibrosis. Furthermore, FTZ ameliorates the intestinal mucosal barrier and intestinal microbiota disorder in mice with NASH.

## Materials and Methods

### Materials and Reagents

Reagents and kits were purchased/obtained as follows: atorvastatin calcium tablets (Pfizer Inc., Dalian, China); hematoxylin and eosin (H&E) (Biosharp Life Sciences, Hefei, China); Oil Red O (Sigma-Aldrich, St. Louis, MO, USA); alanine aminotransferase (ALT), aspartate aminotransferase (AST), total cholesterol (TC), and TG assay kits (Jiancheng Institute of Biotechnology, Nanjing, China); SYBR Green supermix (Bio-Rad, CA, Berkeley, USA); anti-mouse-horseradish peroxidase (HRP) and anti-rabbit-HRP antibodies (Promega, Madison, WI, USA); anti-CD68 antibodies (Boster Biological Technology Co, Ltd., Wuhan, China); anti-Toll-like receptor (TLR), anti-E-cadherin and anti-peroxisome proliferator–activated receptor (PPAR)-gamma antibodies (Proteintech Group, Inc., Rosemont, PA, USA); anti-adipose triglyceride lipase (ATGL), anti-Smad2/3, and anti-phosphorylated Smad3 (p-Smad3) antibodies (Cell Signaling Technology, Inc., MA, Boston, USA; Affinity Biosciences LTD, OH, Columbus USA); anti-zonula occludens-1 (ZO-1) antibodies (Affinity Biosciences LTD, OH, USA); anti-alpha-smooth muscle actin (α-SMA), anti-β-actin, and anti-glyceraldehyde-3-phosphate dehydrogenase (GAPDH) antibodies (Boster Biological Technology Co, Ltd., Wuhan, China).

### Preparation and Quality Control of FTZ

FTZ was obtained from the First Affiliated Hospital of Guangdong Pharmaceutical University (Guangzhou, China). The preparation of FTZ was consistent with the protocol described previously (22). The quality analysis of the FTZ extract was performed by UPLC-MS/MS as described previously (25).

### Animals and Treatment

The experimental procedures were carried out in accordance with the China Animal Welfare Legislation. C57BL/6 mice (male, 6 weeks of age) were purchased from Beijing Vital River Laboratory Animal Technology Co., Ltd., Beijing, China. All mice were divided into five groups, and nine mice in each group: normal chow diet (NCD; protein, 18.3%; fat, 10.2%; carbohydrates, 71.5%; 1025; HUAFUKANG Bioscience Co., Ltd., Beijing, China) group; high-fat diet (HFD; protein, 14%; fat, 42%; carbohydrates, 44%; cholesterol, 0.2%; TP26304; Trophic Diet, Nantong, China) group; FTZ low-dose treatment (i.g.) group (600 mg/kg FTZ); FTZ high-dose treatment (i.g.) group (1,200 mg/kg FTZ); and atorvastatin treatment (i.g.) group (10 mg/kg ATV). NCD served as control. Mice were fed with an HFD for 20 weeks to induce NASH. FTZ and atorvastatin were diluted by 0.5% sodium carboxymethylcellulose (CMC-Na). HFD-fed mice were administrated intragastrically FTZ and atorvastatin since the 11th week for additional 10 weeks. NCD and the HFD vehicle groups were administrated intragastrically with 0.5% CMC-Na during the whole period of experiment.

### Glucose Tolerance Tests

Glucose tolerance test (GTT) and insulin tolerance test (ITT) were performed in mice after an overnight fast. Blood glucose concentration was measured after 0, 15, 30, 45, 60, 90 and 120 min with a glucometer.

### Serum Assays

Serum TG, TC, high-density lipoprotein (HDL), low-density lipoprotein (LDL), ALT, and AST levels were measured by a commercial kit according to the manufacturers’ instruction (Jiancheng Bioengineering Institute, Nanjing, China).

### Quantitative Analysis of Hepatic Triglyceride

Hepatic TG was extracted from liver tissues with a mixture of chloroform and methanol. The contents of hepatic TG were measured by a commercial kit (Jiancheng Bioengineering Institute, Nanjing, China) and normalized by the liver wet weight.

### Analysis of Body Composition

Lean and fat mass were determined by the EchoMRI body composition analyzer (EchoMRI™, Shanghai, China) in mice according to the manufacturers’ instruction.

### Histopathology

Liver and ileum tissues were routinely fixed in 4% paraformaldehyde solution (4°C) overnight, embedded in paraffin, and then sectioned (4 μm) for H&E staining. Lipid droplets were detected by Oil Red O staining in frozen liver sections. Frozen livers were embedded in optical coherence tomography (OCT) and divided into 12 μm sections. All sections were stained by 0.3% Oil Red O for 15 min and hematoxylin for 2 min. Picrosirius red (PSR, 26357-02; Hede Biotechnology Co., Ltd., Beijing, China) staining was carried out to visualize the degree of liver fibrosis. The positive areas were quantified using the Image J. Histological images of section tissues were captured with a light microscope (Olympus, Tokyo, Japan).

### Immunohistochemistry

Liver specimens fixed in 4% paraformaldehyde solution were embedded in paraffin blocks. Liver sections (4 μm thick) were processed using a standard immunostaining protocol. For immunohistochemical analyses, liver sections were separated, rehydrated, and sequentially incubated with primary antibodies and secondary antibodies. The area of positive staining was measured in high-power fields on each slide and quantified using Image J.

### NAFLD Activity Scores

NAFLD activity scores (NASs) are a semi-quantitative scoring system. The main contents of NASs are hepatocyte steatosis, lobular inflammation, and hepatocyte ballooning. The total NASs are the sum of the three scores. 1) Hepatocyte steatosis: 0 (<5%); 1 (5%–33%); 2 (34%–66%); 3 (>66%). 2) Lobular inflammation: 0 (none); 1 (<2); 2 (2–4); 3 (>4). 3) Hepatocyte ballooning: 0 (none); 1 (rare); 2 (common).

### Western Blot Analysis

Tissues were homogenized in radioimmunoprecipitation (RIPA) lysis buffer. The supernatant was collected after centrifugation at 12,000 rpm for 30 min at 4°C. Total proteins (20–40 µg) were electrophoresed on sodium dodecyl sulfate–polyacrylamide gel electrophoresis (SDS-PAGE) gels and transferred to polyvinylidene fluoride membranes (Millipore, MA, Boston, USA; Bio-Rad, CA, Berkeley, USA; New Life Science Products, NY, New York City, USA). Afterwards, the membranes were blocked with 10% nonfat dry milk, followed by incubation with primary and secondary antibodies. Membranes were detected by Clarity Western electrochemiluminescence (ECL) Substrate (Bio-Rad, USA) in conjunction with a chemiluminescence system (New Life Science Products, USA).

### Quantitative Real-Time RT-PCR

The total RNA of liver or ileum tissues was extracted using a TRIzol reagent (Invitrogen, MA, Waltham, USA) and then subjected to reverse transcription and quantitative real-time PCR (qPCR). From the extracted mRNA, cDNA was synthesized using a PrimeScript™ RT reagent kit with gDNA Eraser (Takara, Beijing, China). All the sequence information was shown in **Supplementary Table 1**. Quantitative real-time PCR measurements were performed using the SYBR Green Supermix (Bio-Rad, CA, Berkeley, USA). The primers used were described in **Supplementary Table 1**. The relative amount of each mRNA was calculated by using the comparative threshold cycle (Ct) method. Ct values were normalized to gapdh.

### 16S rRNA Gene Sequencing

In this study, 16S rRNA gene sequencing was used to extract microbial DNA from mice feces, and V3 and V4 were selected for amplification to detect the variation and relative abundance of the target region and to obtain information on the bacterial population for analysis. The PCR reaction system was configured by taking 30 ng of qualified genomic DNA samples and the corresponding fusion primers, setting the corresponding PCR reaction parameters for PCR amplification, using Agencourt AMPure XP magnetic beads to purify the PCR amplification products and dissolve them in an elution buffer to complete the library construction. The libraries were tested for fragment range and concentration using an Agilent 2100 Bioanalyzer. Libraries that pass the assay are sequenced on the appropriate platform (HiSeq/iSeq) depending on the insert size. The data are filtered to remove the low-quality reads, and the remaining high-quality clean data can be used for later analysis; the reads are stitched into tags by the overlap relationship between the reads; the tags are clustered into operational taxonomic units (OTUs) at a given similarity, and then, the OTUs are compared with the database, and they are annotated with species; based on the OTUs and species annotation results, the complexity of the sample species and the species differences between groups were analyzed.

### UPLC-QTOF-MS-Based Metabolomics

To clarify the changes produced by the metabolites of the intestinal flora of NASH mice after treatment with FTZ, we selected three representative samples from the HFD and FTZ (600 mg/kg) groups for a metabolomic study among the mice feces samples in the 16S rRNA gene sequencing by combining the results of previous experiments. The sample and extract solution (acetonitrile:methanol:water = 2:2:1) (1,000 μl) containing the internal standard (L-2-chlorophenylalanine, 2 μg/ml) were mixed, then homogenized, sonicated, and centrifuged. The supernatant was dried in a vacuum concentrator. The samples were reconstituted in 200 μl of 50% acetonitrile and centrifuged for 10 min, and the supernatant was ready for UPLC-MS/MS analysis. The UPLC and MS/MS instrument settings are shown in **Supplementary Tables 2, 3**. The UPLC separation was carried out using a 1290 Infinity series UPLC System (Agilent Technologies, CA, Palo Alto, USA).

### Statistical Analysis

All data were presented as mean ± standard deviation (mean ± SEM) and statistically analyzed using Graphpad Prism 8.0 and SPSS 19.0 software. The differences between the two groups were compared using a t-test. A Mann–Whitney U test or Wilcox. test was used for two groups with different variances. The differences between multiple groups were compared using one-way ANOVA. Pathway-associated metabolite sets Small Molecule Pathway Database (SMPDB) were calculated by a hypergeometric test to obtain the P-value. *p* < 0.05 indicates a statistically significant difference.

## Results

### FTZ Ameliorates HFD-Induced Obesity and Insulin Resistance in Mice

**Figure 1:**
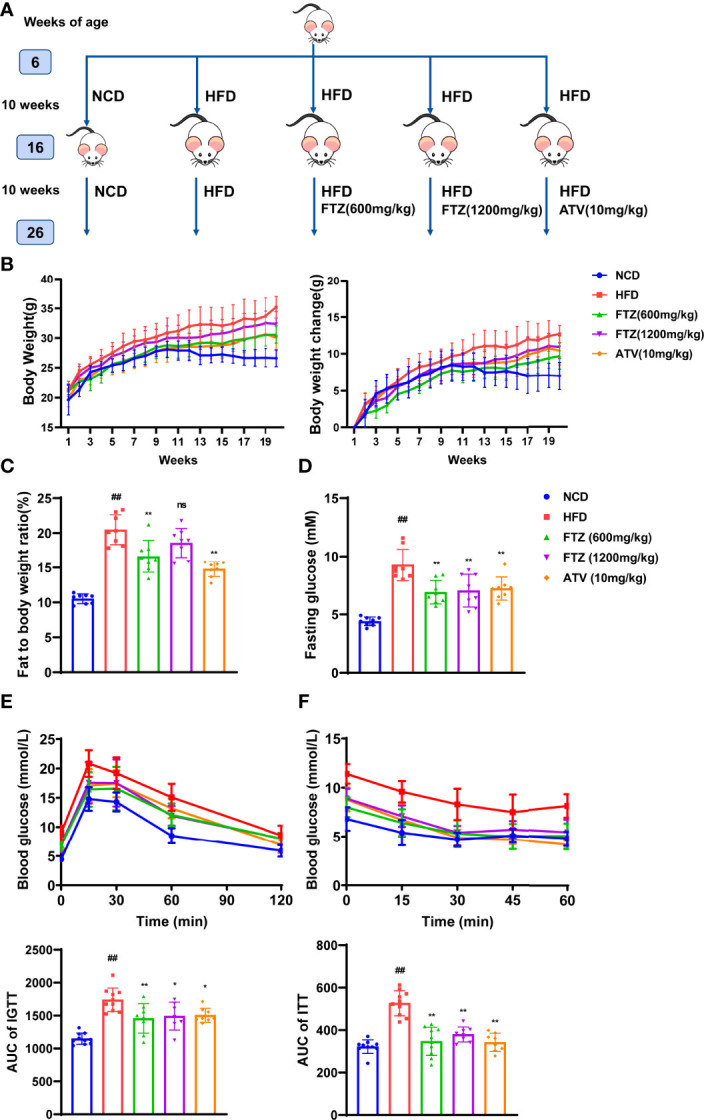
Experimental methods using FTZ and the effects of FTZ on weight gain, obesity, and insulin sensitivity in HFD-induced obese mice. **(A)** The experimental approach. **(B)** Fasting body weight of mice at week 20 (n=8-10). **(C)** Fat-to-body weight ratio (n=8). **(D)** Fasting insulin (n=8). **(E, F)** The IPGTT and IPITT assays were performed to evaluate the insulin sensitivity of mice in the indicated groups treated with NCD, HFD, or FTZ (n=7-10). Data are represented as means ± SEM. # indicates a significant difference between the NCD group and the HFD group (t-test); * indicates a significant difference between the FTZ (600 mg/kg)/FTZ (1,200 mg/kg)/ATV (10 mg/kg) group and the HFD group (one-way ANOVA). ^##^ *P* < 0.01 versus NCD mice; ^*^ *P* < 0.05, ^**^ *P* < 0.01 versus mice fed by HFD. ns indicates no significance.

The occurrence of NASH is closely related to systemic metabolic disorders such as obesity and insulin resistance (26). To determine the therapeutic effects of FTZ on NASH, we first investigated whether FTZ administration affected the systemic metabolic state. Mice with NASH were induced by HFD for 20 weeks (**Figure 1A**). Body weight was significantly increased in HFD-fed mice compared with NCD, whereas FTZ and ATV-treated mice had less weight gain than HFD-fed mice administrated with vehicle (**Figure 1B**). Similarly, FTZ and ATV treatment reduced the total fat mass in HFD-fed mice (**Figure 1C**). These data suggested that both FTZ and ATV treatment alleviated obesity in HFD-fed mice. Furthermore, FTZ treatment decreased the fasting blood glucose in HFD-fed mice (**Figure 1D**). GTT and ITT assays showed that FTZ and ATV markedly alleviated impaired glucose intolerance and insulin resistance induced by HFD (**Figures 1E, F**). Collectively, these data suggest that FTZ attenuates obesity and insulin resistance in mice with NASH.

### FTZ Attenuates Hepatic Steatosis and Lipotoxic Injury Induced by HFD in Mice

**Figure 2:**
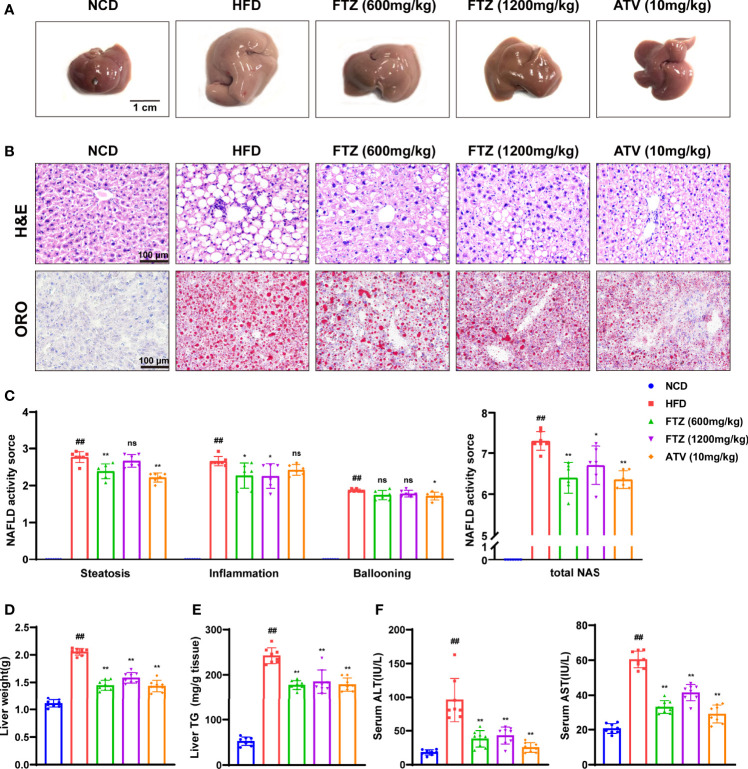
Effects of FTZ on hepatic steatosis and lipotoxicity in mice. **(A)** Representative macroscopic images of the livers of NCD, HFD, FTZ, and ATV mice. **(B)** Representative images of H&E and oil red O staining of liver sections. **(C)** NAS score in the indicated groups (n=6-7). **(D)** Liver weight (n=8). **(E)** Liver TG content (n=8). **(F)** Serum ALT and AST levels of the mice in the indicated groups (n=8). Data are represented as means ± SEM. # indicates a significant difference between the NCD group and the HFD group (t-test); * indicates a significant difference between the FTZ (600 mg/kg)/FTZ (1,200 mg/kg)/ATV (10 mg/kg) group and the HFD group (one-way ANOVA). ^##^ *P* < 0.01 versus NCD mice; ^*^ *P* < 0.05, ^**^ *P* < 0.01 versus mice fed by HFD. ns indicates no significance.

The size and weight of the livers from HFD-fed mice were significantly increased compared with those in the NCD group, while they were significantly reversed by FTZ treatment (**Figures 2A, D**). Liver morphology analysis showed that notable hepatic lipid deposition was observed in HFD-fed mice, whereas it was significantly attenuated in the FTZ treatment group. Additionally, NAS scores also indicated that FTZ treatment ameliorated liver lipotoxicity and injury in the mice induced by HFD. Oil red O staining also showed that the density of hepatic lipid droplets as well as the size of the lipid droplets was obviously decreased by FTZ treatment compared to those of HFD-fed mice (**Figures 2B, C**). Moreover, the upregulation of hepatic TG, a key indicator of causing hepatic lipotoxicity, was significantly rolled back by FTZ (**Figure 2E**). In addition, FTZ attenuated the upregulation of serum ALT and AST in mice caused by HFD feeding (**Figure 2F**). Furthermore, FTZ alleviated the abnormal serum levels of TG, TC, HDL, and LDL caused by HFD (**Figures S1A-D**). These data suggest that FTZ attenuates HFD-induced hepatic steatosis and lipotoxic injury in mice.

### FTZ Normalizes Hepatic Lipid Metabolism Genes in Mice Fed With HFD

**Figure 3:**
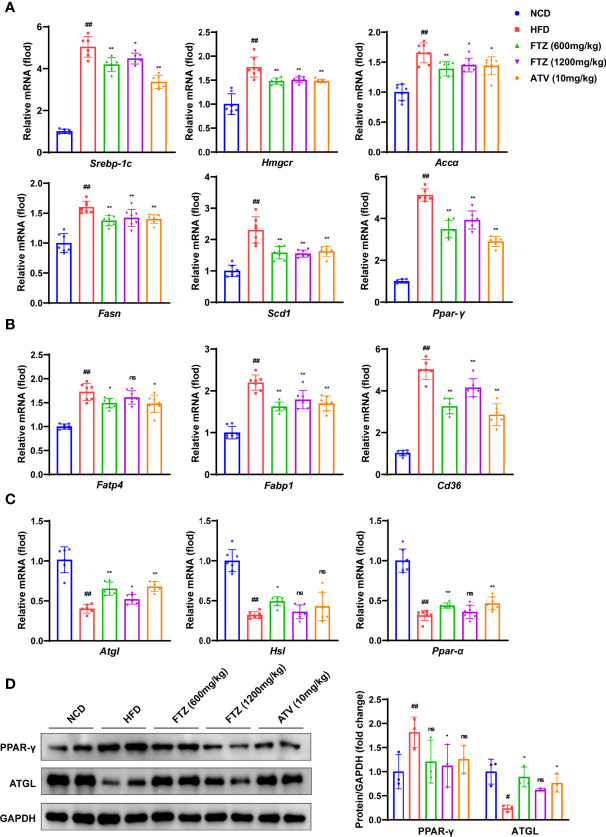
Effects of FTZ on liver lipid metabolism in mice. **(A–C)** The mRNA expression levels of genes associated with fatty acid synthesis, transport, lipolysis, and β-oxidation (n=6-7). **(D)** The expression of PPAR-γ, ATGL, and GAPDH was analyzed by Western blotting. GAPDH served as a loading control (n=3). Data are represented as means ± SEM. # indicates a significant difference between the NCD group and the HFD group (t-test); * indicates a significant difference between the FTZ (600 mg/kg)/FTZ (1,200 mg/kg)/ATV (10 mg/kg) group and the HFD group (one-way ANOVA). ^#^ *P* < 0.05, ^##^ *P* < 0.01 versus NCD mice; ^*^ *P* < 0.05, ^**^ *P* < 0.01 versus mice fed by HFD. ns indicates no significance.

To verify the role of FTZ in hepatic lipid metabolism, we examined the genes involved in lipid biosynthesis, lipolysis, and uptake. Indeed, the mRNA levels of lipogenic genes such as *Srebp-1c, Hmgcr, Accα, Fasn, Scd1*, and *Ppar-γ* were increased by HFD treatment, whereas they were decreased by FTZ and ATV treatment (**Figure 3A**). In addition, FTZ and ATV treatment reduced the mRNA levels of hepatic lipid transport genes such as *Fatp4, Fabp1*, and *Cd36* in HFD-fed mice (**Figure 3B**). Conversely, the hepatic mRNA levels of oxidative phosphorylation genes such as *Atgl, Hsl*, and *Ppar-α* were decreased by HFD, whereas they were upregulated by FTZ and ATV treatment (**Figure 3C**). Immunoblotting analysis confirmed that the protein expression of ATGL and PPAR-γ was markedly restored in mice treated by FTZ or ATV, compared with mice fed by HFD (**Figure 3D**). These data suggested that FTZ ameliorates the disorder of hepatic lipid metabolism induced by HFD in mice.

### FTZ Attenuated Liver Metabolic Inflammation and Progressive Fibrosis Induced by HFD in Mice

**Figure 4:**
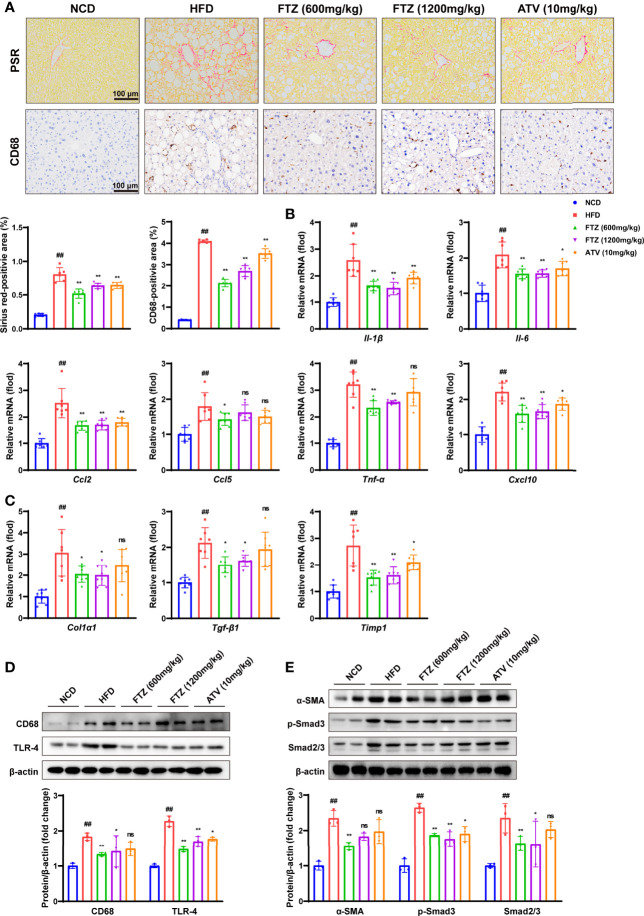
Effects of FTZ on metabolic inflammation and fibrosis in the livers of mice. **(A)** Representative images showing PSR and immunohistochemical staining of CD68 staining in the livers of the indicated mice (n=6). **(B)** Relative mRNA levels of inflammatory genes in the livers of the indicated mice (n=7–8). **(C)** Relative mRNA levels of profibrotic genes in the livers of the indicated mice (n=7-8). **(D)** The expression of CD68, TLR-4, and β-actin was analyzed by Western blotting. β-actin served as a loading control (n=3). **(E)** The expression of α-SMA, p-Smad3, Smad2/3, and β-actin was analyzed by Western blotting. β-actin served as a loading control (n=3). Data are represented as means ± SEM. # indicates a significant difference between the NCD group and the HFD group (t-test); * indicates a significant difference between the FTZ (600 mg/kg)/FTZ (1,200mg/kg)/ATV (10 mg/kg) group and the HFD group (one-way ANOVA). ^##^ *P* < 0.01 versus NCD mice; ^*^ *P* < 0.05, ^**^ *P* < 0.01 versus mice fed by HFD. ns indicates no significance.

To further investigate the protective effects of FTZ against NASH *in vivo*, we examined the effects of FTZ on NASH-associated inflammation and fibrosis. The activation of hepatic stellate cells (HSCs) and collagen deposition are important distinctions between NAFL and NASH (27). Consistently, picrosirius red (PSR) staining showed less collagen deposition in the liver sections of FTZ treatment. Furthermore, FTZ attenuated hepatic inflammatory cell infiltration, as demonstrated by CD68 immunohistochemistry staining (**Figure 4A**). The q-PCR assay showed that FTZ treatment significantly attenuated the increase in the hepatic mRNA levels of inflammatory genes such as *Il-β, Il-6, Tnf-α, Cxcl10, Ccl2*, and *Ccl5* (**Figure 4B**). Additionally, FTZ attenuated the hepatic mRNA levels of profibrotic genes such as *Col1a1, Tgf-β1*, and *Timp1* in NASH mice (**Figure 4C**). The protein levels of CD68 and TLR4 were significantly downregulated by FTZ treatment (**Figure 4D**). Furthermore, the expression of α-SMA, a marker of activated hepatic stellate cells, was also attenuated by FTZ treatment (28). In parallel, the phosphorylation of Smad2/3 was suppressed in FTZ-treated mice (**Figure 4E**). These data suggested that FTZ attenuated hepatic steatosis, inflammation, and fibrosis in mice under a metabolic stress condition.

### FTZ Restores Intestinal Mucosal Barrier Disruption and Inflammation Induced by HFD in Mice

**Figure 5:**
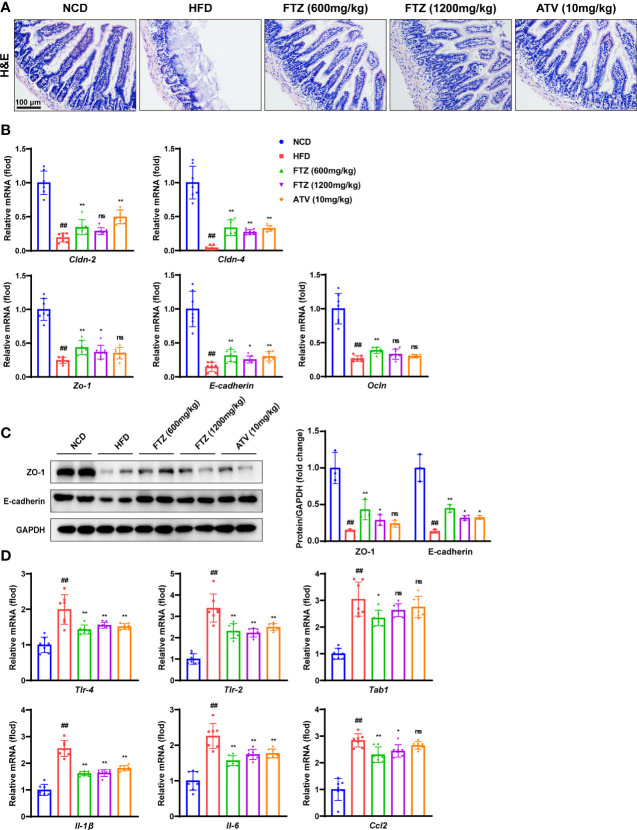
Effects of FTZ on intestinal mucosal barrier and inflammation in mice. **(A)** Representative images of H&E staining of ileum sections. **(B)** Relative mRNA levels of ileum tight junction molecule genes of the indicated mice (n=7). **(C)** The expression of ZO-1, E-cadherin, and GAPDH was analyzed by Western blotting. GAPDH served as a loading control (n=3). **(D)** Relative mRNA levels of ileum inflammation genes of mice in the indicated group (n=7). Data are represented as means ± SEM. # indicates a significant difference between the NCD group and the HFD group (t-test); * indicates a significant difference between the FTZ (600 mg/kg)/FTZ (1,200 mg/kg)/ATV (10 mg/kg) group and the HFD group (one-way ANOVA). ^##^ *P* < 0.01 versus NCD mice; ^*^ *P* < 0.05, ^**^ *P* < 0.01 versus mice fed by HFD. ns indicates no significance.

Increasing evidence demonstrated that increased gut inflammation and compromised intestinal epithelial permeability promote the enhanced translocation of a multitude of gut microbial products involved in the progression of NAFLD (29). Next, the H&E staining of intestinal histopathology showed that intestinal mucosal barrier disruption and inflammation induced by HFD were effectively mitigated by FTZ and ATV treatment (**Figure 5A**). Epithelial tight junction molecules such as ZO-1, occludin, claudin 4 and claudin 2, and E-cadherin, are the markers of the epithelium integrity (30). As expected, the mRNA levels of *Cldn-2*, *Cldn-4*, *Zo-1, E-cadherin*, and *Ocln* were significantly downregulated in HFD-fed mice compared with those in NCD-treated mice (**Figure 5B**). In contrast, FTZ and ATV treatment significantly reversed the decrease in these tight junction molecules in HFD-fed mice. Immunoblotting analysis confirmed that the protein expression of ZO-1 and E-cadherin was markedly restored in mice treated by FTZ or ATV, compared with mice fed by HFD (**Figure 5C**). Moreover, intestinal inflammatory cell infiltration was markedly increased in NASH mice but remarkably decreased by FTZ and ATV treatment. The q-PCR assay showed that FTZ and ATV treatments significantly attenuated the HFD-induced interstitial mRNA levels of inflammatory genes such as *Tlr4*, *Tlr2*, *Tab1*, *Il-1b*, *Il-6*, and *Ccl2* in mice (**Figure 5D**). With regard to the gut–liver axis, abnormal lipid metabolism in the intestine may lead to excessive lipid flow into the portal circulation, resulting in more hepatic lipid accumulation (13). As expected, FTZ significantly reduced the abundance of ileum lipid transport–related genes induced by HFD, such as *Cd36*, *Fabp1*, *Fatp4*, and *Ffar3* (**Figure S2**). Collectively, these data suggested that FTZ treatment protected mice against intestinal mucosal barrier disruption and inflammation induced by HFD treatment.

### FTZ Reverses the Differences in the Composition of the Intestinal Flora of HFD-Induced NASH Mice

**Figure 6:**
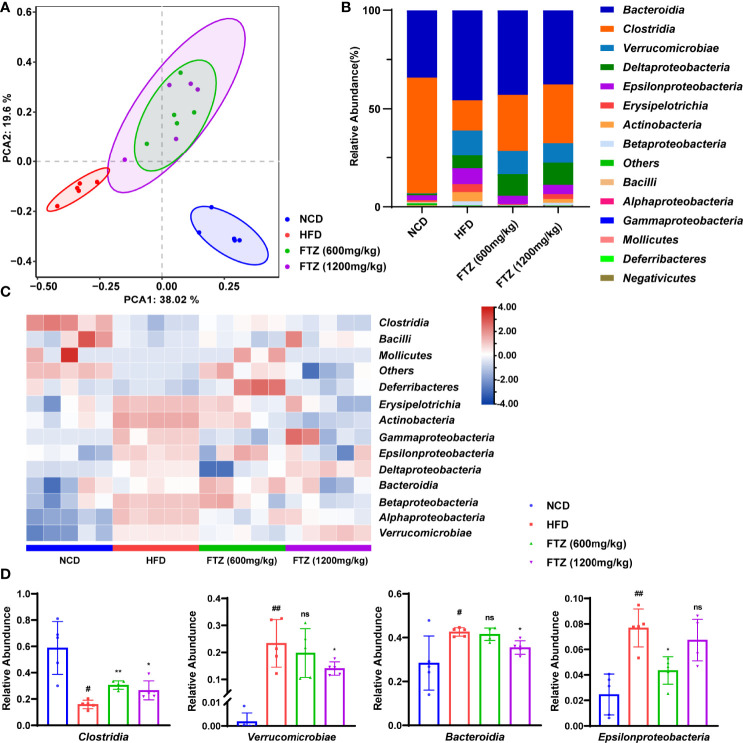
Effects of FTZ on the intestinal flora compartment of mice. **(A)** PCA score plot (n=5). **(B)** Average class distribution of gut microbiomes. **(C)** The heat map (clustered at class level) showing that the flora was significantly altered by HFD and FTZ treatment compared to NCD (n=5). **(D)** The comparison of the taxonomic abundance of the indicated groups (n=5). Data are represented as means ± SEM. # indicates a significant difference between the NCD group and the HFD group (t-test); * indicates a significant difference between the FTZ (600 mg/kg)/FTZ (1,200 mg/kg) group and the HFD group (one-way ANOVA). ^#^ *P* < 0.05, ^##^ *P* < 0.01 versus NCD mice; ^*^ *P* < 0.05, ^**^ *P* < 0.01 versus mice fed by HFD. ns indicates no significance.

Strategies to restore the intestinal microbiota to prevent and cure metabolic diseases have been proposed (31). To better understand the complex interactions between FTZ treatment and gut microbiota in NASH mice, the phylogenetically informative 16S rRNA genes (high-variability regions V3–V4) were amplified from the total DNA extracted from mouse fecal samples. The principal component analysis (PCA) clearly separated the samples from the NCD-fed mice, HFD-fed mice, and FTZ-treated mice. (**Figure 6A**). Furthermore, the separation of non-metric multidimensional scaling (NMDS) and the principal coordinate analysis (PCoA) of the weighted UniFrac was consistent with PCA (**Figures S3A-B**). The calculated relative abundance of bacteria at the class level (top 15) in the five groups is shown in **Figure 6B**. The intestinal flora composition of NASH mice was changed compared with control mice. Especially, the abnormal elevation of class *Bacteroidia*, *Verrucomicrobiae*, and *Epsilonproteobacteria* in NASH mice was significantly attenuated by FTZ treatment. In contrast, the decrease in *Clostridia* from NASH mice was significantly increased by FTZ treatment (**Figures 6C, D**). Consistently, a similar recovery was observed at the family level of the flora (**Figure S3C**). In addition, the abnormal reduction of the difference in alpha diversity in NASH mice was significantly attenuated by FTZ treatment (**Figures S3D, E**). These data suggested that FTZ alleviated the gut microbiota disorder in NASH mice.

### FTZ Treatment Exhibits Different Intestinal Flora Metabolites From HFD-Induced NASH Mice

**Figure 7:**
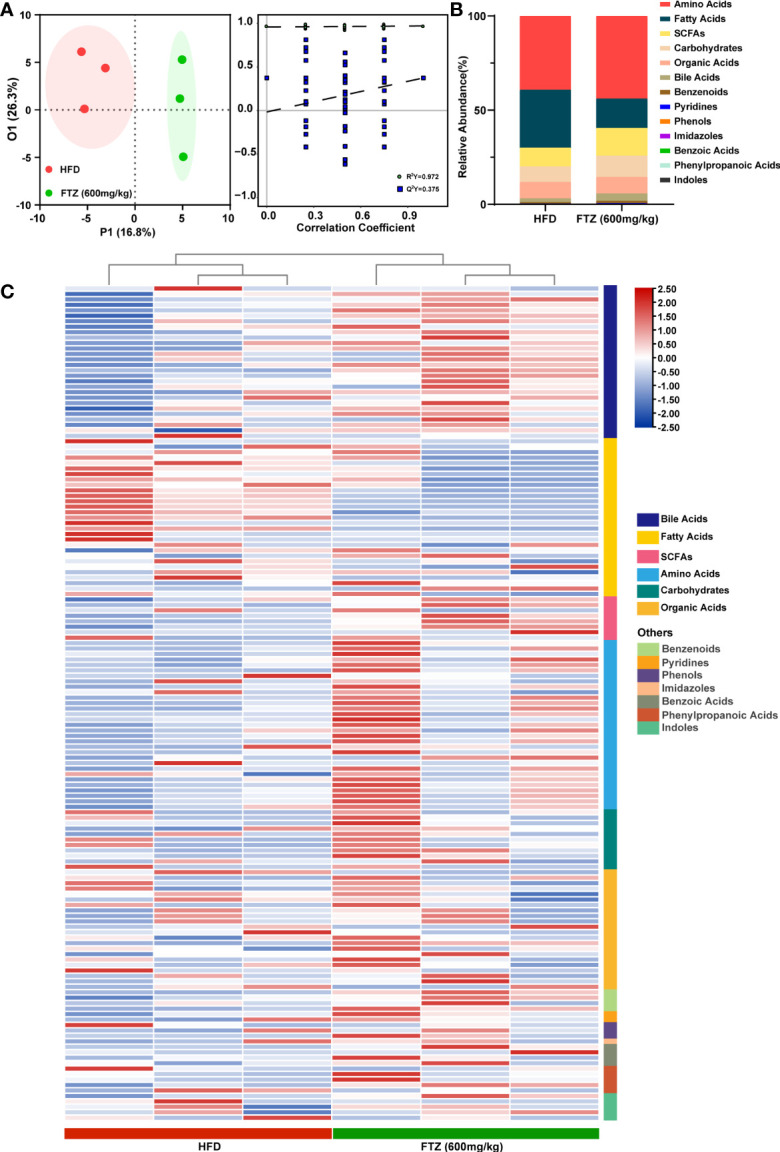
Effects of FTZ on the metabolite composition of the intestinal flora of mice. **(A)** OPLS-DA 2D score chart and permutation test (n=3). **(B)** Relative abundance statistics for the median values of each metabolite in each group of samples. **(C)** Z-score heat map for the overall metabolite profile (n=3).

Dramatic differences in intestinal metabolites were demonstrated between NCD and HFD mice by metabolomics analysis, suggesting that intestinal microbiota readily mediated the pathogenesis of NASH in mice (**Supplementary Figures 4A–C**). To further analyze the differences in metabolic profiles between the FTZ treatment group and the HFD group, a supervised discriminant model, orthogonal partial least squares discriminant analysis (OPLS-DA), was used to perform the analysis. Based on the OPLS-DA model, score plots and the permutation test illustrated an obvious separation between the HFD and FTZ groups (**Figure 7A**). A statistical stacked histogram of the relative abundance of medium values for each metabolite category in each group of samples demonstrates the significant differences in intestinal flora metabolites, including amino acids, fatty acids, bile acids, and SCFAs, between the HFD and FTZ treatment groups (**Figure 7B**). In contrast, the Z-score heat map shows the trend in the concentration of each class of metabolites in the samples of NASH mice versus FTZ-treated mice. The results showed that the FTZ group of mice had greater differences in the content of intestinal flora metabolites compared to the HFD group, with higher levels of bile acids, fatty acids, and SCFAs overall, as well as greater variability (**Figure 7C**). The above results indicate that the intestinal metabolites of NASH mice appear significantly different after FTZ treatment compared to HFD, suggesting the presence of potential biomarkers.

### Metabolite Profile Associated With Bile Acid Metabolism Was Mediated by FTZ Treatment in NASH Mice

**Figure 8:**
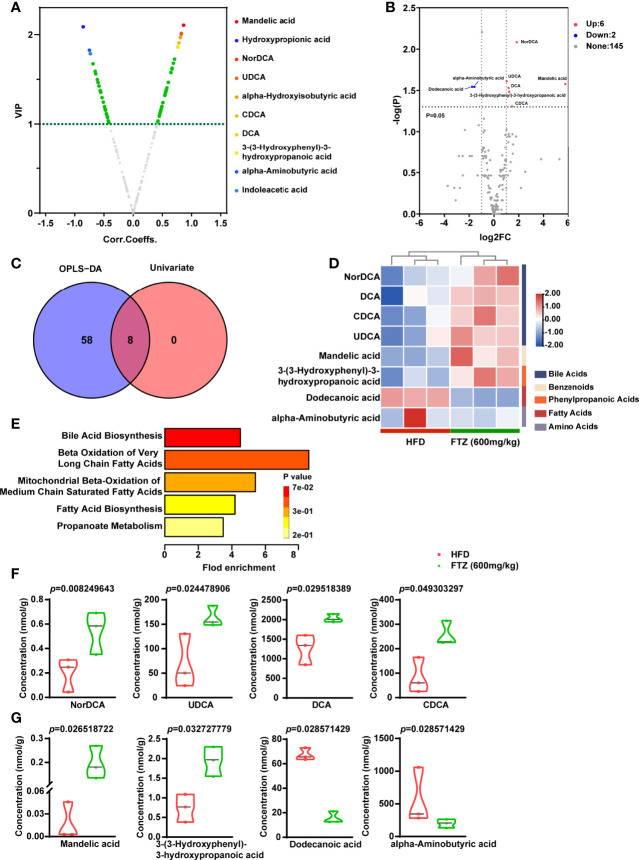
Effects of FTZ on the potential biomarkers of intestinal metabolites in mice. **(A)** Volcano map based on OPLS-DA model results (VIP) > 1. **(B)** Volcano plot based on a one-dimensional test with the threshold set as follows: 1) *P* < 0.05 (t-test or Mann–Whitney U test); 2) absolute value of log2FC >1. **(C)** Venn diagram and the corresponding scores of the differential metabolites based on the data from the OPLS-DA model and one-dimensional test. **(D)** Z-score heat map of potential biomarkers (n=3). **(E)** Results of the pathway enrichment analysis of differential metabolites using selected SMPDB libraries (hypergeometric test). **(F–J)** Unidimensional statistical analysis of violin plots for the top 8 differential metabolites in terms of p-value (t-test or Wilcox. test; n=3).

Taking the merges or intersections of differential metabolites obtained unidimensionally and multidimensionally can be used to select potential biomarkers that may have a biological significance based on unidimensional and multidimensional analysis. Based on the results of the OPLS-DA model, the volcano plot was used to screen for reliable metabolic markers. The results showed that a total of 66 metabolites met Variable importance in projection (VIP) > 1 in the HFD compared to the FTZ treatment group, and the top 10 differential metabolites have been color-coded (**Figure 8A**). We also used a unidimensional test (We selected the t-test or Mann–Whitney U test based on the normality and chi-square of the data) to obtain the differential metabolites between the two groups mentioned above. The results showed that a total of eight differential metabolites were obtained from this unidimensional analysis (**Figure 8B**). For the combined analysis of the two tests above, there were 8 identical differential metabolites in the OPLS-DA and unidimensional analyses; a further 58 differential metabolites were present only in the OPLS-DA results, and 0 differential metabolites were present only in the unidimensional analysis results (**Figure 8C**). The Z-score heat maps for the eight metabolites that met the screening criteria showed significant differences with NASH mice after FTZ treatment: NorDCA, UDCA, DCA, CDCA, mandelic acid, and 3-(3-hydroxyphenyl)-3-hydroxypropanoic acid were elevated, while alpha-aminobutyric acid and dodecanoic acid were decreased (**Figure 8D**). We used the differential metabolites obtained that met the screening criteria for pathway enrichment analysis. The pathway enrichment analysis of differential metabolites using selected SMPDB libraries showed multiple pathways including bile acid metabolism, the beta oxidation of very long chain fatty acids, mitochondrial beta oxidation of medium-chain saturated fatty acids, fatty acid biosynthesis, and propanoate metabolism (**Figure 8E**). Finally, the absolute concentrations of the above potential biomarkers were measured in the samples to verify and obtain results consistent with the previous section (We selected the t-test or Wilcox. test based on the normality and chi-square of the data) (**Figures 8F-J**).

## Discussion

In the present study, we demonstrated that the FTZ treatment markedly ameliorated NASH in mice. In addition to its commonly accepted protective effect on hepatic lipid metabolism, metabolic inflammation, and progressive fibrosis, we found that the mechanisms by which FTZ alleviated NASH are involved in the attenuation of gut inflammation and microbiota disorders, as well as the improvement of intestinal barrier function. Lifestyle modification is beneficial for the treatment of NAFLD and should be managed on a long-term basis (32). However, poor adherence to lifestyle modification makes NAFLD management a difficult task. Unfortunately, there are no specific drugs to date for the treatment of NASH (33).

FTZ is a clinical formula created by Professor GJ, a renowned Chinese herbalist, based on the theory of “Tiaogan Qishu Huazhuo Rule,” which has been used to treat hyperlipidemia and metabolic syndrome, and related complications such as atherosclerosis and non-alcoholic fatty liver disease. “Tiaogan Qishu Huazhuo Rule” offers its distinctive treatment options based on the “differentiation of symptoms and signs.” The complexity of the material basis of Chinese medicine determines the multiplicity of its effects, which leads to the diversity of its medicinal properties (34). In a recent study, some components of FTZ were found to prevent the development of fatty liver in rats (22, 23). In the present study, we have demonstrated that FTZ alleviates metabolic stress–induced NASH by regulating the gut–liver axis and preventing steatosis, inflammation, and fibrosis. Furthermore, FTZ is more effective than ATV in combating metabolic inflammation and progressive fibrosis, which may be attributed to the large number of active ingredients in it, such as berberine and oleanolic acid (35, 36).

Our data are consistent with previous studies that FTZ has a hypolipidemic effect under metabolic stress conditions, and its therapeutic effect is similar to that of ATV, which is recommended for NAFLD treatment in several national clinical guidelines, including the United States, and can effectively reduce lipid deposition and lipotoxicity caused by HFD (37–39). Our results revealed that FTZ-regulated hepatic fatty acid synthesis by the suppression of fatty acid synthesis regulators such as FAS, acetyl-CoA carboxylase (ACC), and SREBP-1c, suggesting that FTZ may limit hepatic TG availability by inhibiting lipogenesis. FTZ also regulates a wide range of genes involved in lipid catabolism and transport. PPAR-γ expression is increased in high-fat diet–induced hepatic lipid accumulation, and the hepatic deficiency of PPAR-γ expression inhibits HFD-induced NAFLD progression in obese mice (40, 41). Meanwhile, PPAR-γ directly regulates CD36 transcript levels, thus promoting hepatic lipid uptake and affecting lipid metabolism, resulting in hepatic lipid deposition and steatosis in NAFLD (42, 43). In this study, our data showed that FTZ alleviated the expression of PPAR-γ and CD36 in livers from HFD-fed mice at least in part, which may impact the severity of hepatic steatosis as well as improve whole-body insulin sensitivity.

As a hallmark of the pathogenesis of NASH, the inflammatory response involves two critical events: the release of numerous types of proinflammatory cytokines and damage to hepatocytes (44). In terms of liver inflammation, the number of CD68-positive cells reflected macrophage recruitment; the mRNA levels of inflammatory factors such as *Il-1β, Il-6, Ccl2, Ccl5, Cxcl10*, and *Tnf-α* were significantly increased in response to HFD diet induction (45–47). TLR4 is capable of triggering the rapid activation of its downstream signaling, NF-κB, which upregulates the production of proinflammatory cytokines such as IL-1β and IL-6. Previous studies have extensively reported the TLR signaling pathway as a key target to communicate with the liver and intestine (48, 49). The infectious agent/LPS-mediated activation of TLR leads to the release of hepatic inflammatory factors that exacerbate NASH, which is one of the biological bases for FTZ-mediated liver–gut interaction in the treatment of NASH (50).

Hepatic fibrosis is one of the key histopathological features in NASH patients, suggesting a more severe and progressive liver injury (51). The vast majority of drugs developed for NASH have been rejected because they do not reverse progressive fibrosis (9, 52). In the present study, FTZ attenuated collagen deposition and the hepatic expression of profibrotic growth factors in mice fed by the HFD diet, suggesting that FTZ protects against NASH-related fibrosis through the suppression of HSC activation. Interestingly, the protein expression of p-Smad3 and Smad2/3 was significantly attenuated by FTZ treatment. These results suggest that FTZ reduces HFD-induced progressive liver fibrosis in mice by inhibiting the activation of Smad3 pathways and HSCs.

Consistently, a large number of studies, in both NASH patients and animals, have confirmed the involvement of the dysbiosis of the intestinal flora in the progression of NAFLD (53). In this study, we found that the composition of the gut microbiota of FTZ-treated mice was different from that of HFD-fed mice but similar to that of NCD-fed mice. It is noteworthy that in previous reports, the relative proportion of the phylum *Bacillus mimicus* in the intestinal flora of obese individuals was reduced in comparison with slim individuals, and that this proportion recovered after weight loss on a controlled diet (54). However, a clinical study of microbiota composition in NAFLD reported an increased proportion of *Bacteroidetes* in obese and NASH patients compared with healthy controls (55). These species differences may be caused by interspecies metabolic differences between humans and mice, the composition and brand of the diet, and the modeled feeding times. Furthermore, *Epsilonproteobacteria*, named after the Greek god Proteus, is a common pathogenic organism and previous studies have found an increase in *Epsilonproteobacteria* in obese patients and NASH patients compared to healthy controls, and it is reassuring to note that FTZ treatment can counteract these adverse effects of HFD (55). We also observed favorable improvements in the differential strains at the family level of taxonomy consistent with previous reports, such as *Porphyromonadaceae*, *Bacteroidaceae*, *Ruminococcaceae*, and *Verrucomicrobiaceae* (56–58). In addition, there are numerous animal experiments with results consistent with our differential strain changes. Previous studies have noted that the oral administration of branched-chain amino acids (3% kcal) induced a significant increase in *Ruminococcaceae* and portal acetic acid levels, and it reduced hepatic fat accumulation in HFD rats (59). Another NASH study suggested that a Chinese herbal formula reduced hepatic steatosis maybe through decreasing certain gut bacteria (such as *Verrucomicrobiaceae*), alleviating intestinal endotoxemia, and reducing NLRP3 inflammasome activation (60). Supplementation with prebiotic VSL#3 may improve NASH by reducing the relative abundance of *Porphyromonadaceae* and *Bacteroidia* and increasing the relative abundance of *Ruminococcaceae*, thereby reshaping the intestinal flora of NASH mice (61). Our results confirm that FTZ impairs dyslipidemia, hepatic steatosis, and lipotoxicity in NASH mice, and FTZ was found to achieve the remodeling of intestinal flora in NASH mice by affecting the above-mentioned differential strains.

Consistent with expectations, as FTZ restored homeostasis to the intestinal flora of NASH mice, it also produced significant changes in the composition of their metabolites. We subsequently identified eight potential biomarkers by taking intersections between univariate analysis and multivariate analysis. Bile acid–related pathways may be involved given that the gut microbiota, through defined enzymatic activities (such as deconjugation, dehydroxylation, oxidation, and epimerization, among others), is a critical modulator of the pool size and composition of bile acids and can significantly modify the chemical and signaling properties of bile acids (62, 63). Of the differential strains we identified for the FTZ treatment of NASH, *Clostridium*, *Bacteroides*, and *Ruminococcaceae* were reported to be involved in the metabolic process of bile acids (64, 65). The role of bile acid metabolism in regulating glucose and lipid metabolism is well established (58, 66, 67). Interestingly, we found four bile acids (CDCA, UDCA, NorDCA, and DCA) from the eight differential metabolites. Of these, the primary bile acid CDCA has been reported as a natural ligand for FXR, confirming its involvement in lipid metabolism, immunomodulation, and anti-inflammatory protection (68). Furthermore, UDCA is considered a potential metabolite for the treatment of NASH because of its anti-inflammatory, anti-apoptotic, and antioxidant properties (69). In another study of Chinese herbal compounds for the treatment of NASH, NorDCA was also observed to increase in the feces of mice (70). In addition, a human study showed that serum cholesterol levels decreased in every subject (an average of 15%) during DCA administration (71).

LC/MS-based metabolomics has led to new biomarker discoveries and a better mechanistic understanding of FTZ for NASH. However, many major pertinent challenges remain. Partial, incomplete metabolomes persist due to factors such as limitations in mass spectrometry data acquisition speeds, a wide range of metabolite concentrations, and intestinal flora-specific changes that confound our understanding of metabolite perturbations (72, 73). Since LC/MS-based metabolomics data usually varied greatly, it should be cautious and validated on making conclusions based on only three samples. Larger samples are required for metabolic analysis in future studies to make the data and conclusion more convincing.

**Figure 9:**
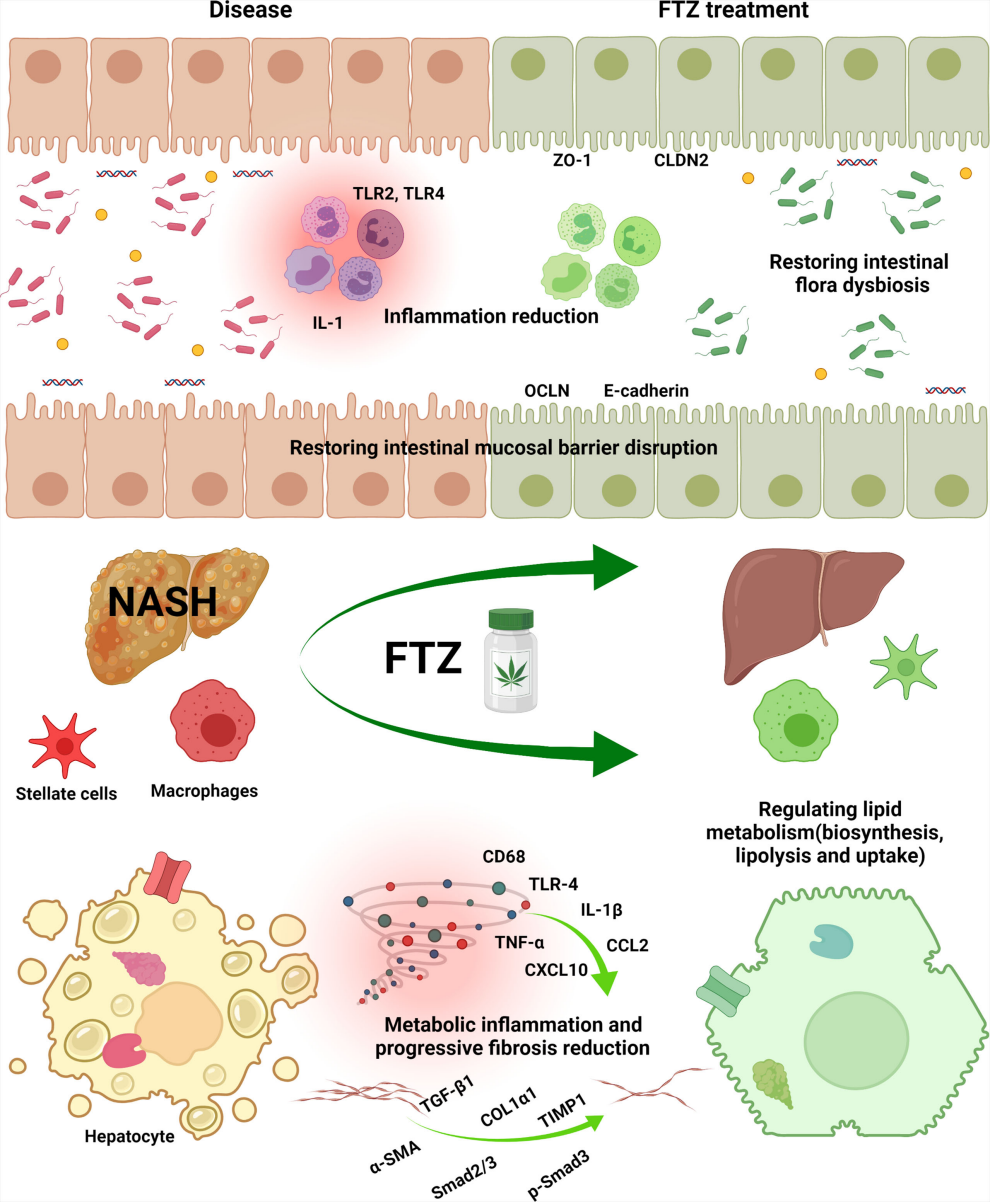
FTZ attenuated HFD-induced hepatic steatosis, inflammatory responses, and fibrosis in mice. FTZ reduces lipotoxicity by regulating hepatic lipid synthesis, transport, and catabolism. Moreover, FTZ alleviates NASH and progressive liver fibrosis by inhibiting hepatic inflammation, stellate cell activation, and collagen deposition. These protective effects are achieved by modulating tight junctions, restoring intestinal barrier function, and improving the dysregulation of intestinal flora and its metabolites in mice.

Collectively, our current findings demonstrate that FTZ ameliorates the pathogenesis of NASH by inhibiting hepatic lipid accumulation, inflammation, and fibrogenesis while improving intestinal flora disruption and barrier function disruption. Therefore, our findings will provide insights into the development of Chinese medicine treatments for NASH (**Figure 9**).

## Conclusion

In conclusion, our current study provides compelling evidence that FTZ can attenuate lipid deposition, metabolic inflammation, and hepatic fibrosis in NASH, possibly by modulating intestinal bacterial microbes and their metabolic homeostasis. FTZ may be a potential candidate for the treatment of patients with NASH.

## Data Availability Statement

The datasets presented in this study can be found in online repositories. The names of the repository/repositories and accession number(s) can be found below: https://www.ncbi.nlm.nih.gov/, SRP350756.

## Ethics Statement

The animal study was reviewed and approved by Research Ethical Committee of Guangdong Pharmaceutical University.

## Author Contributions

JG conceived and designed the experiments. TL, TX, YF, and XL carried out the experiments and wrote the manuscript. SJ, ZY, LP, and XR took part in the discussion and proofreading the manuscript. All authors have reviewed and approved the final version of the manuscript.

## Funding

This work was supported by grants from the National Natural Science Foundation of China (81830113, 81870420 and 82070590), the Major Basic and Applied Basic Research Projects in Guangdong Province of China (2019B030302005), the National Key R & D Plan of China "Research on Modernization of Traditional Chinese Medicine" program (2018YFC1704200) and the Special Topics of General Projects of Guangzhou Science and Technology Plan of China (201904010075).

## Conflict of Interest

The authors declare that the research was conducted in the absence of any commercial or financial relationships that could be construed as a potential conflict of interest.

## Publisher’s Note

All claims expressed in this article are solely those of the authors and do not necessarily represent those of their affiliated organizations, or those of the publisher, the editors and the reviewers. Any product that may be evaluated in this article, or claim that may be made by its manufacturer, is not guaranteed or endorsed by the publisher.

## Abbreviations

ACCα, acetyl-CoA carboxylase**;** ALT, alanine aminotransferase**;** AST, aspartate aminotransferase**;** ATGL, adipose triglyceride lipase**;** ATV, atorvastatin**;** CCL2, C-C motif chemokine ligand 2**;** CCL5, C-C motif chemokine ligand 5**;** CD36, fatty acid translocase CD36**;** CLDN, claudin**;** CMC-Na, sodium carboxymethylcellulose**;** CXCL5, C-x-c motif chemokine ligand 5**;** CXCL10, C-x-c motif chemokine ligand 10**;** FAA, free fatty acid**;** FABP1, fatty acid-binding protein 1**;** FAS, fatty acid synthase**;** FATP4, fatty acid transport protein 4**;** FFAR3, free fatty acid receptor 3**;** FTZ, Fufang Zhenzhu Tiaozhi capsule**;** GTT, glucose tolerance test**;** HDL, high-density lipoprotein**;** HFD, high-fat diet**;** HRP, horseradish peroxidase**;** HSCs, hepatic stellate cells**;** HSL, hormone-sensitive lipase**;** IL-6, interleukin-6**;** IL-1β, interleukin-1β**;** IR, insulin resistance**;** ITT, insulin tolerance test**;** LDL, low-density lipoprotein**;** NAFLD, nonalcoholic fatty liver disease**;** NAS, NAFLD activity score**;** NASH, non-alcoholic steatohepatitis**;** NCD, normal chow diet**;** NF-κB, nuclear factor-κB**;** NMDS, non-metric multidimensional scaling**;** OTUs, operational taxonomic units**;** PCA, principal component analysis**;** PCoA, principal coordinates analysis**;** PPAR-α, peroxisome proliferator–activated receptor α**;** p-Smad3, phosphorylated smad3**;** PSR, picrosirius red**;** qPCR, quantitative real-time PCR**;** SCD1, stearoyl-CoA desaturase 1**;** SREBP-1c, sterol regulatory element-binding protein 1c**;** TC, total cholesterol**;** TCM, traditional Chinese medicine**;** TG, triglyceride**;** TGF-β1, transforming growth factor-β1**;** TLR, Toll-like receptor**;** TNF-α, tumor necrosis factor-α**;** ZO-1, zonula occludens-1**;** α-SMA, alpha-smooth muscle actin.
