# Supplementary material for: Fufang Zhenzhu Tiaozhi Capsule Prevents Intestinal Inflammation and Barrier Disruption in Mice With Non-Alcoholic Steatohepatitis
Source: Front Endocrinol (Lausanne). 2022 Jun 16;13:864703. doi: 10.3389/fendo.2022.864703 (PMC9243428; doi:10.3389/fendo.2022.864703)
Supplement: Supplementary file 1 [file DataSheet_1.docx]

Supplementary Material

**Supplementary Table 1 Primer sequences for qPCR**

| **Genes** | **Forward Primers** | **Reverse Primers** |
| --- | --- | --- |
| *Srebp-1c* | CGACTACATCCGCTTCTTGCAG | CCTCCATAGACACATCTGTGCC |
| *Hmgcr* | CACAATAACTTCCCAGGGGT | GGCCTCCATTGAGATCCG |
| *Accα* | GTTCTGTTGGACAACGCCTTCAC | GGAGTCACAGAAGCAGCCCATT |
| *Fasn* | CACAGTGCTCAAAGGACATGCC | CACCAGGTGTAGTGCCTTCCTC |
| *Scd1* | GCAAGCTCTACACCTGCCTCTT | CGTGCCTTGTAAGTTCTGTGGC |
| *Ppar-γ* | GCATGGTGCCTTCGCTGA | TGGCATCTCTGTGTCAACCATG |
| *Fatp4* | CACGAATCAGAACAGAGAGGC | TGCTTTGGTTTCTGGGACTT |
| *Fabp1* | GATTTCTGACACCCCCTTGA | TGCAGAGCCAGGAGAACTTT |
| *Cd36* | TGAGACTGGGACCATTGGTGAT | CCCAAGTAAGGCCATCTCTACCAT |
| *Atgl* | GAGGAATGGCCTACTGAACCA | GGCTGCAATTGATCCTCCTCT |
| *Hsl* | AGGGAGGGCCTCAGCG | TTGGCTGGTGTCTCTGTGTC |
| *Ppar-α* | GCAGGAGATCTACAAGGACTTG | CCCTCAGAATAGTGCAACTGG |
| *Il-1β* | TTGTTGATGTGCTGCTGTGA | TGTGAAATGCCACCTTTTGA |
| *Il-6* | GAAATGATGGATGCTACCAAACTG | GACTCTGGCTTTGTCTTTCTTGTT |
| *Tnfα* | GGTCTGGGCCATAGAACTGA | CAGCCTCTTCTCATTCCTGC |
| *Cxcl10* | ATCATCCCTGCGAGCCTATCCT | GACCTTTTTTGGCTAAACGCTTTC |
| *Ccl2* | GCTACAAGAGGATCACCAGCAG | GTCTGGACCCATTCCTTCTTGG |
| *Ccl5* | CCTGCTGCTTTGCCTACCTCTC | ACACACTTGGCGGTTCCTTCGA |
| *Col1a1* | GCTCCTCTTAGGGGCCACT | CCACGTCTCACCATTGGGG |
| *Tgf-β1* | GGCCAGATCCTGTCCAAGC | GTGGGTTTCCACCATTAGCAC |
| *Timp1* | CTGTTGGCTGTGAGGAATGC | CGGGACTGGAAGCCCTTTTC |
| *Tlr4* | TGAGGACTGGGTGAGAAATGAGC | CTGCCATGTTTTGAGCAATCTCAT |
| *Tlr2* | TTCACCACTGCCCGTAGATG | TCTACTGTGATTCGCTTCGCCTTC |
| *Tab1* | CAGGCGGATTGGGGATTAC | GGCAAACTCGGTGTCAATCAT |
| *Zo-1* | GGGAAAACCCGAAACTGATG | GCTGTACTGTGAGGGCAACG |
| *Occludin* | CCCAGGCTTCTGGATCTATGT | TCCATCTTTCTTCGGGTTTTCA |
| *Claudin4* | TGATTATGGTGCCCGTGTCC | CGAGTAGGGCTTGTCGTTGC |
| *Claudin2* | GTGACGTCCAGTGCAATGTC | ATGCCATGAAGATTCCAAGC |
| *E-cadherin* | TCTGGCAC-CACACCTTCTAC | GGAAGGAAGGCT-GTAAGAGT |
| *Hmgcr* | CACAATAACTTCCCAGGGGT | GGCCTCCATTGAGATCCG |
| *Gapdh* | AGGAGCGAGACCCCACTAACA | AGGGGGGCTAAGCAGTTGGT |

**Supplementary Table 2 UPLC instrument settings**

| **UPLC Parameters** | **instrument settings** |
| --- | --- |
| Column | ACQUITY UPLC BEH C18 1.7 μM VanGuard pre-column (2.1×5 mm) and analytical column (2.1 × 100 mm) |
| Column Temp. (°C) | 40 |
| Sample Manager Temp. (°C) | 10 |
| Mobile Phases | A=water with 0.1% formic acid; and B=acetonitrile /IPA (70:30) |
| Gradient Conditions | 0-1 min (5% B), 1-11min (5-78% B), 11-13.5 min (78-95% B), 13.5-14 min (95-100% B), 14-16 min (100% B)，16-16.1 min (100-5% B)，16．1-18 min (5% B) |
| Flow Rate (mL/min) | 0.40 |
| Injection Vol. (μl) | 5.0 |

**Supplementary Table 3 MS/MS instrument settings**

| **Parameters** | **instrument settings** |
| --- | --- |
| Capillary (Kv) | 1.5 (ESI+), 2.0 (ESI-) |
| Source Temp (°C) | 150 |
| Desolvation Temp (°C) | 550 |
| Desolvation Gas Flow (L/Hr) | 1000 |

## Supplementary Figures


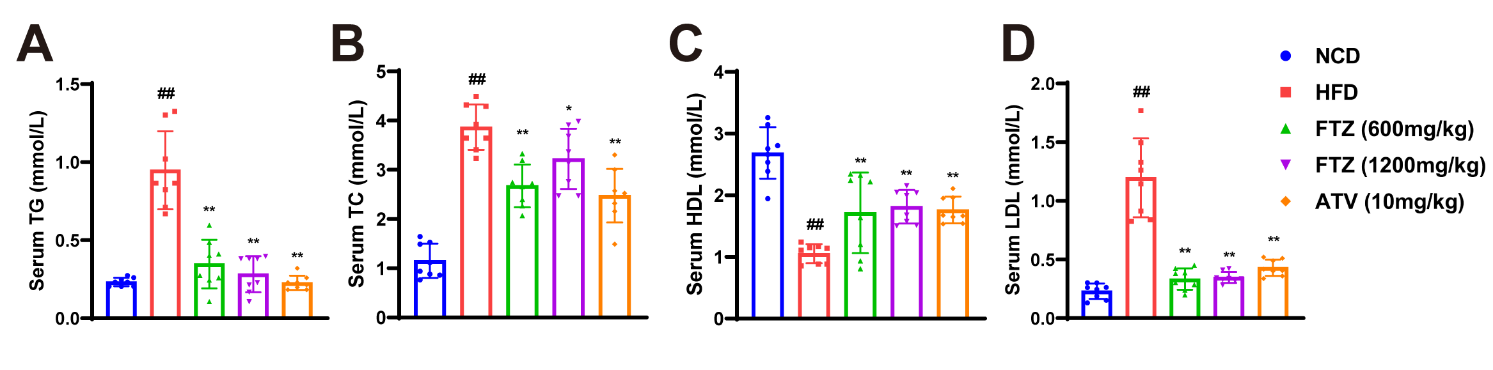


**Supplementary Figure 1. Effects of FTZ on serum biochemical parameters in NASH mice.**

(A) The serum contents of TG of mice in the indicated groups (n=7-8). (B) The serum contents of TC of mice in the indicated groups (n=7-8). (C) The serum contents of HDL of mice in the indicated groups (n=8). (D) The serum contents of LDL of mice in the indicated groups (n=8). Data are represented as means ± SEM. # indicates a significant difference between the NCD group and the HFD group (t-test); *indicates a significant difference between the FTZ (600mg/kg)/ FTZ (1200mg/kg)/ ATV (10mg/kg) group and the HFD group (one-way ANOVA). ^##^*P* < 0.01 versus NCD mice; ^*^*P* < 0.05, ^**^*P* < 0.01 versus mice fed by HFD.


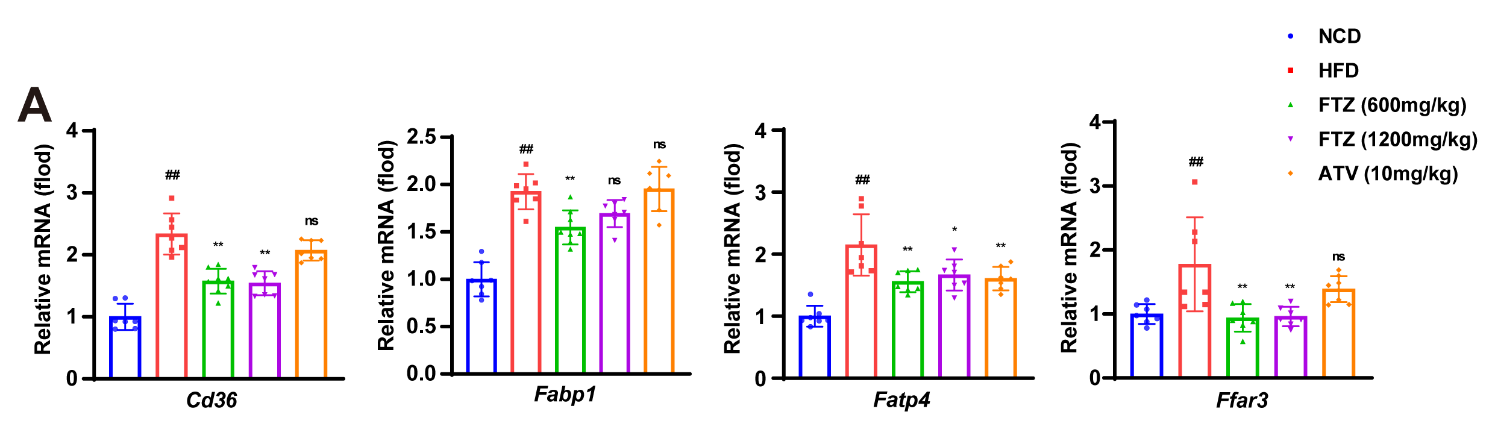
**Supplementary Figure 2.** **Effects of FTZ on the mRNA levels of intestinal lipid transport genes in NASH mice.**

Relative mRNA levels of ileum lipid transport genes in the indicated mice (n=7). Data are represented as means ± SEM. # indicates a significant difference between the NCD group and the HFD group (t-test); *indicates a significant difference between the FTZ (600mg/kg)/ FTZ (1200mg/kg)/ ATV (10mg/kg) group and the HFD group (one-way ANOVA). ^##^*P* < 0.01 versus NCD mice; ^*^*P* < 0.05, ^**^*P* < 0.01 versus mice fed by HFD; “ns” stands for not significant. ns indicates no significance. ns indicates no significance.


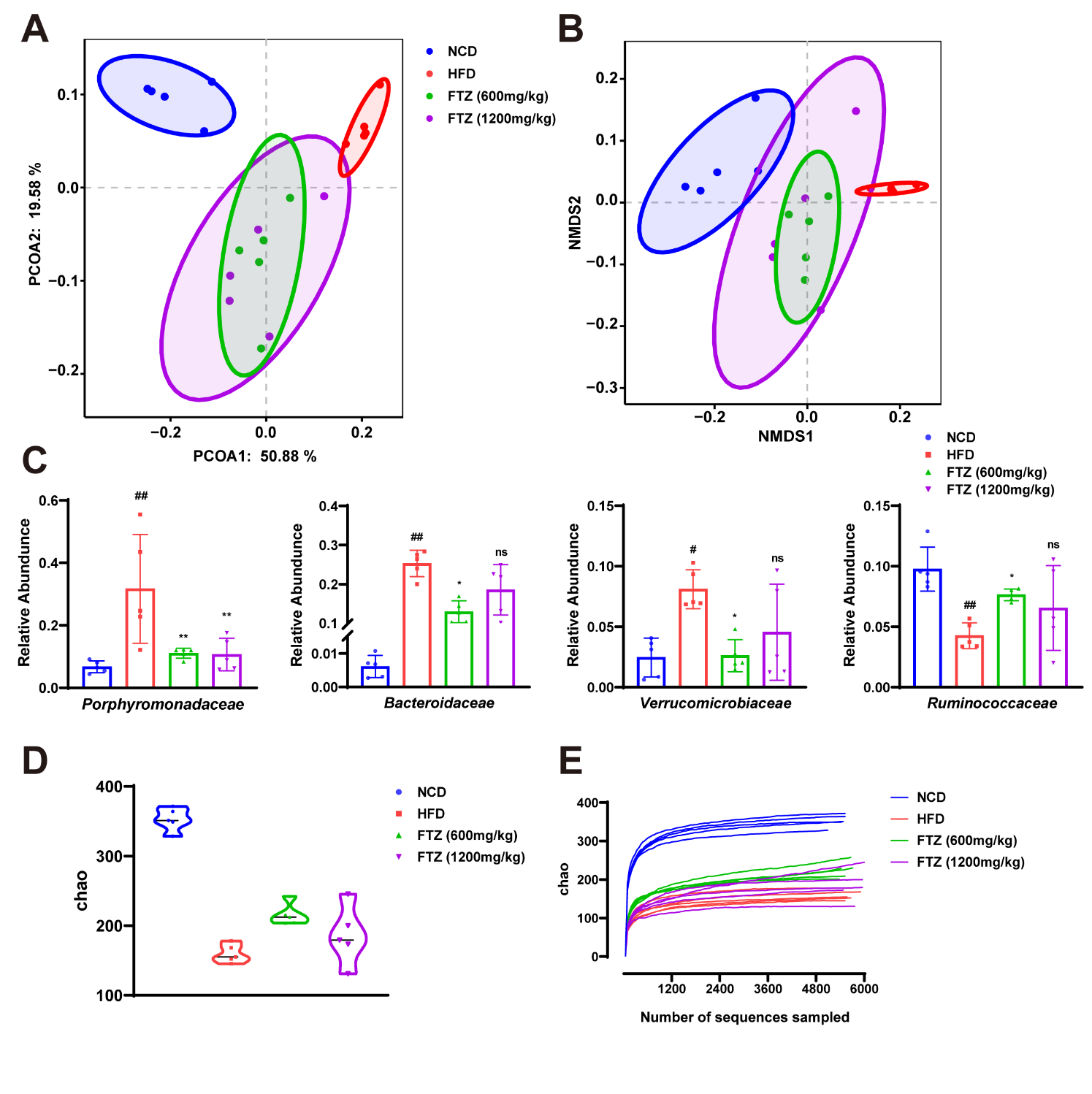


**Supplementary Figure 3.** **Effects of FTZ on the diversity and structure of the intestinal flora of NASH mice.**

(A) PCoA score plot (weighted UniFrac metric) (n=5). (B) NMDS score plot (n=5). (C) The comparison of the taxonomic abundance among the indicated groups (n=5). (D) Intergroup differences in alpha diversity (n=5). (E) Alpha Diversity rarefraction curve (chao diversity index) (n=5). Data are represented as means ± SEM. # indicates a significant difference between the NCD group and the HFD group (t-test); *indicates a significant difference between the FTZ (600mg/kg)/ FTZ (1200mg/kg) group and the HFD group (one-way ANOVA). ^#^*P* < 0.05, ^##^*P* < 0.01 versus NCD mice; ^*^*P* < 0.05, ^**^*P* < 0.01 versus mice fed by HFD; “ns” stands for not significant. ns indicates no significance.


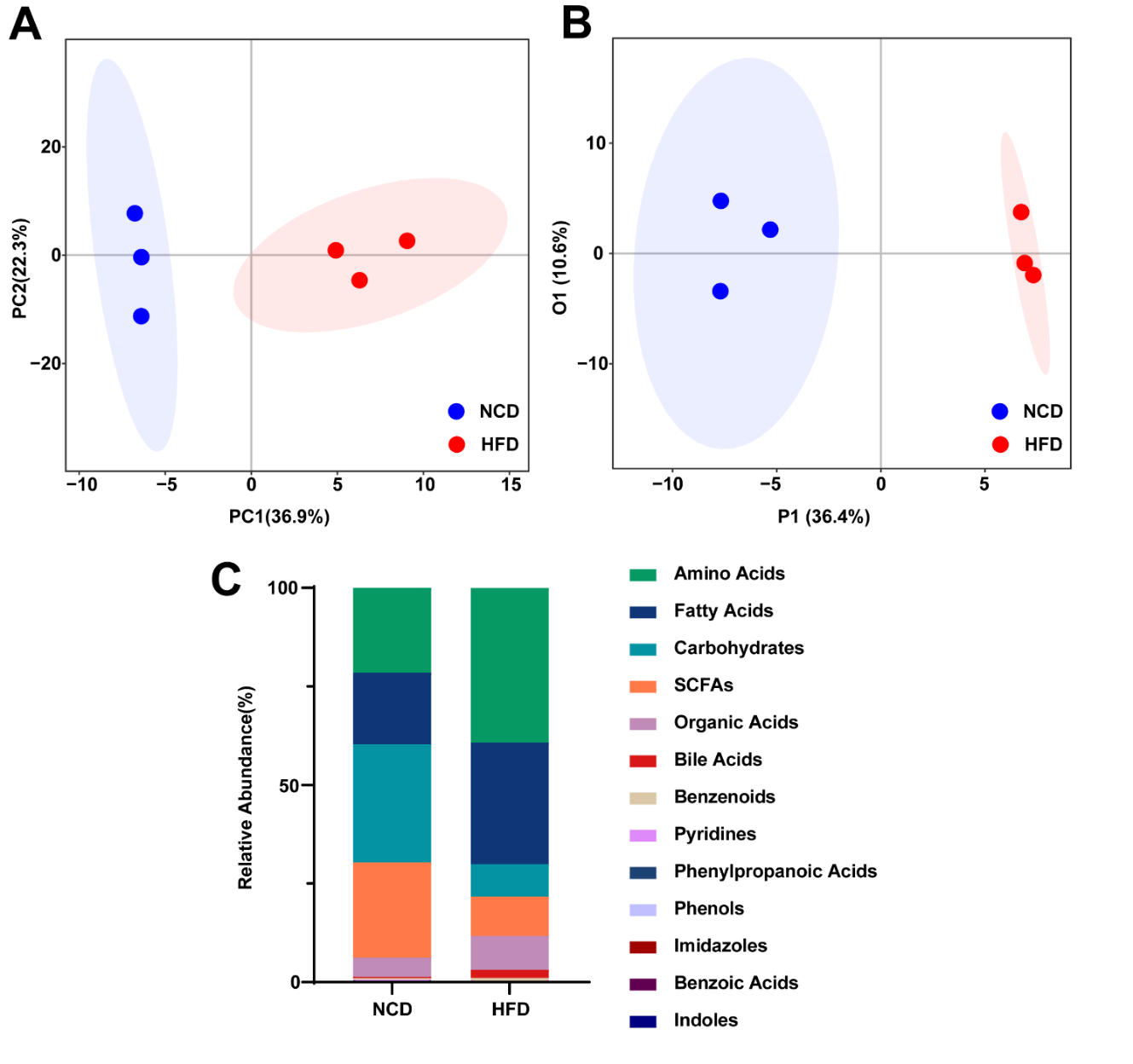


**Supplementary Figure 4. Effects of HFD on the metabolite composition of the intestinal flora of mice.**

(A) PCA 2D score chart (n=3). (B) OPLS-DA 2D score chart (n=3). (C) Relative abundance statistics for median values of each metabolite in each group of samples.
